# Peri-operative antibiotics acutely and significantly impact intestinal microbiota following bariatric surgery

**DOI:** 10.1038/s41598-020-77285-7

**Published:** 2020-11-23

**Authors:** Harika Nalluri, Scott Kizy, Kristin Ewing, Girish Luthra, Daniel B. Leslie, David A. Bernlohr, Michael J. Sadowsky, Sayeed Ikramuddin, Alexander Khoruts, Christopher Staley, Cyrus Jahansouz

**Affiliations:** 1 Department of Surgery, University of Minnesota, 11-132 Phillips-Wangesteen Bldg, 516 Delaware Street SE, Minneapolis, MN 55455 USA; 2 CentraCare Bariatric Center, 1200 Sixth Ave N, St. Cloud, MN 56303 USA; 3 Park Nicollet Bariatric Surgery Center, 3931 Louisiana Ave S, Minneapolis, MN 55426 USA; 4 Department of Biochemistry, Molecular Biology and Biophysics, University of Minnesota, 6-155 Jackson Hall, 321 Church St SE, Minneapolis, MN 55455 USA; 5 BioTechnology Institute, University of Minnesota, 140 Gortner Lab, 1479 Ave, St. Paul, MN 55108 USA; 6 Division of Gastroenterology, Department of Medicine, University of Minnesota, 420 Delaware Street SE, MMC 36, Minneapolis, MN 55455 USA; 7 Division of Colon and Rectal Surgery, Department of Surgery, University of Minnesota, 420 Delaware St. SE, Minneapolis, MN 55455 USA

**Keywords:** Microbiome, Dysbiosis, Metabolic syndrome, Obesity

## Abstract

Bariatric surgery is the most effective treatment for weight loss. Vertical sleeve gastrectomy (VSG) involves the resection of ~ 80% of the stomach and was conceived to purely restrict oral intake. However, evidence suggests more complex mechanisms, particularly postoperative changes in gut microbiota, in facilitating weight loss and resolving associated comorbidities. VSG in humans is a complex procedure and includes peri-operative antibiotics and caloric restriction in addition to the altered anatomy. The impact of each of these factors on the intestinal microbiota have not been evaluated. The aim of this study was to determine the relative contributions of each of these factors on intestinal microbiota composition following VSG prior to substantial weight loss. Thirty-two obese patients underwent one of three treatments: (1) VSG plus routine intravenous peri-operative antibiotics (n = 12), (2) VSG with intravenous vancomycin chosen for its low intestinal penetrance (n = 12), and (3) caloric restriction (n = 8). Fecal samples were evaluated for bacterial composition prior to and 7 days following each intervention. Only patients undergoing VSG with routine peri-operative antibiotics showed a significant shift in community composition. Our data support the single dose of routine peri-operative antibiotics as the most influential factor of intestinal microbial composition acutely following VSG.

## Introduction

Despite recent advances in the medical treatment for obesity and T2DM, bariatric surgery remains the most effective therapy resulting in metabolic improvement prior to substantial weight loss^1–3^*.* Vertical sleeve gastrectomy (VSG), a procedure involving the resection of ~ 80% of the stomach, is now the most prevalent form of bariatric surgery in the United States^4^*.* Although VSG was conceived as a purely restrictive surgery to limit oral intake, recent evidence suggests more complex underlying mechanisms, particularly postoperative changes in the composition of the intestinal microbiota, which facilitate weight loss and counteract important comorbidities such as T2DM. Understanding the mechanisms by which bariatric surgery alters physiology is likely to enable alternative therapies, enhance primary results of surgical intervention, and reduce failures^2,5^.

Intestinal microbiota plays a central role in energy metabolism and gut functioning, and have been shown to influence obesity^6^. Transfer of intestinal microbiota from genetically obese mice, or obese human individuals, into germ-free mice results in greater fat mass and increased peripheral insulin resistance^6,7^. Observations from mouse models of both VSG and Roux-en-Y gastric bypass (RYGB) have shown that the procedures are associated with an acute and sustained shift in the composition of the microbiota, a shift that persists longitudinally^8–11^. These observations have been seen in parallel human studies as well, and appear to correlate with the resolution of T2DM^12–14^. Taken a step further, germ-free mice colonized with stool from patients who have undergone bariatric surgery exhibit reduced adiposity suggesting a mechanistic role^14^.

While animal studies are performed in a controlled setting, bariatric surgery in humans is a very complex intervention that includes caloric restriction and peri-operative antibiotics in addition to the anatomic alteration. These factors represent significant confounders in investigating changes in the intestinal microbiota. Post-operatively, bariatric patients are routinely placed on caloric restriction in the form of a liquid diet for at least 1 week following surgery^15^. Modifications in diet, particularly when as drastic as that followed by patients post-operatively, can acutely impact gut microbiota composition^16^. All bariatric surgery patients also receive peri-operative antibiotics that provide surgical site infection prophylaxis and reduce rates of post-surgical wound infections^17^. However, pertinent to metabolic disease, animal models suggest that antibiotics may also contribute to increased adiposity and altered levels of hormones related to glucose and lipid metabolism^18^. Furthermore, we have shown in a mouse model of VSG that short-term antimicrobial administration that disrupt the intestinal microbial community structure not only diminish weight loss following surgery, but have the potential to eliminate any metabolic benefit^19^. The roles that each of these factors play in shaping the composition of intestinal microbiota following surgery have not been investigated and remain relatively unknown. Characterizing the impact of antibiotics and caloric restriction on intestinal microbial composition is important not only for the broader understanding of the physiologic consequences of these interventions but particularly important in the bariatric setting in which the ultimate goal is maximizing weight loss and metabolic benefit.

Our goal was to evaluate the relative contributions of each of these factors in shaping the gut microbiome acutely following VSG in order to reduce confounding and ultimately identify changes that may contribute to the metabolic efficacy of the procedure. To do so, we studied the microbiota and fecal bile acid composition in three groups of patients prior to surgery and on post-operative day 7. Specifically, we examined patients: (1) undergoing VSG with routine peri-operative intravenous (IV) antibiotics consisting of cefazolin or clindamycin (RVSG); (2) undergoing VSG with IV vancomycin (VVSG) as the peri-operative antibiotic; (3) placed on a calorically restricted diet (CR) to approximate the post-operative intake following surgery. Intravenous vancomycin was specifically chosen as a substitute for routine antibiotics because it is predicted to have limited penetration into the gut lumen^20^.

## Results

### Subject demographics

There were no baseline differences in age, weight, BMI, hemoglobin A1c (HbA1c), and fasting glucose levels among all three cohorts (Table 1). There were no post-operative complications, and all patients were discharged by post-operative day (POD) 2, tolerating oral intake. Post-operative narcotic use was also similar between surgical groups. All three cohorts lost a similar amount of weight by POD 7. A 3-month follow-up in the two post-VSG cohorts indicated a statistically similar weight loss.

**Table 1:** Demographics of subjects undergoing routine VSG (RVSG), VSG with IV Vancomycin (VVSG) or caloric restriction (CR).

	RVSG	VVSG	CR	Statistical significance
Total patients	12	12	8	–
Gender (M/F)	3/9	2/10	1/7	–
Age (years)	39.2 ± 3.0	41.4 ± 2.4	47.4 ± 4.2	NS
Weight at intervention (kg)	123.3 ± 7.4	113.2 ± 2.6	107.8 ± 6.7	NS
1 Week weight loss (% TWL)	2.4 ± 0.4	3.7 ± 0.4	4.2 ± 0.4	NS
3 Month weight loss (% TWL)	11.2 ± 2.4	12.4 ± 2.7	–	NS
BMI (kg/m^2^)	42.6 ± 2.1	41.1 ± 2.9	39.7 ± 1.6	NS
HbA1c (%)	5.9 ± 0.1	5.8 ± 0.2	6.4 ± 0.2	NS
Plasma glucose (mg/dl)	108.5 ± 7.5	98.8 ± 3.1	101.6 ± 3.6	NS

Values represent mean ± SEM.

*TWL *total weight loss, *NS *not significant.

### Bacterial composition of fecal samples

A mean Good’s coverage of 99.4 ± 0.2% was observed among all samples, and 131 to 617 OTUs were obtained from a single sample. No differences in species (alpha) diversity, measured by the Shannon index, were observed in pre-intervention samples among all treatment arms (Table 2). Only bacterial communities in feces of VVSG patients had significantly lower Shannon indices than pre-operative communities (*P* = 0.035). Post-intervention bacterial communities in the RVSG and CR arms had similar alpha diversity to those prior to intervention.

**Table 2:** Coverage and alpha diversity (mean ± standard error) in patient samples prior to and following VSG with routine antibiotics (RVSG), VSG with intravenous Vancomycin (VVSG), or caloric restriction (CR).

Arm	Time point	Coverage (%)	*S_obs_	Shannon
RVSG	Pre	99.3 ± 0.1	421 ± 31	3.82 ± 0.11^A^
	Post	99.5 ± 0.1	342 ± 35	3.43 ± 0.15^A^
VVSG	Pre	99.5 ± 0.0	317 ± 15	3.45 ± 0.15^A^
	Post	99.7 ± 0.0	239 ± 14	2.87 ± 0.15^B^
CR	Pre	99.3 ± 0.1	379 ± 29	3.64 ± 0.08^A^
	Post	99.4 ± 0.0	366 ± 24	3.43 ± 0.19^A,B^

*S_obs_—number of OTUs observed.

^A,B^Values sharing the same superscript did not differ significantly by post-hoc test.

**Figure 1:**
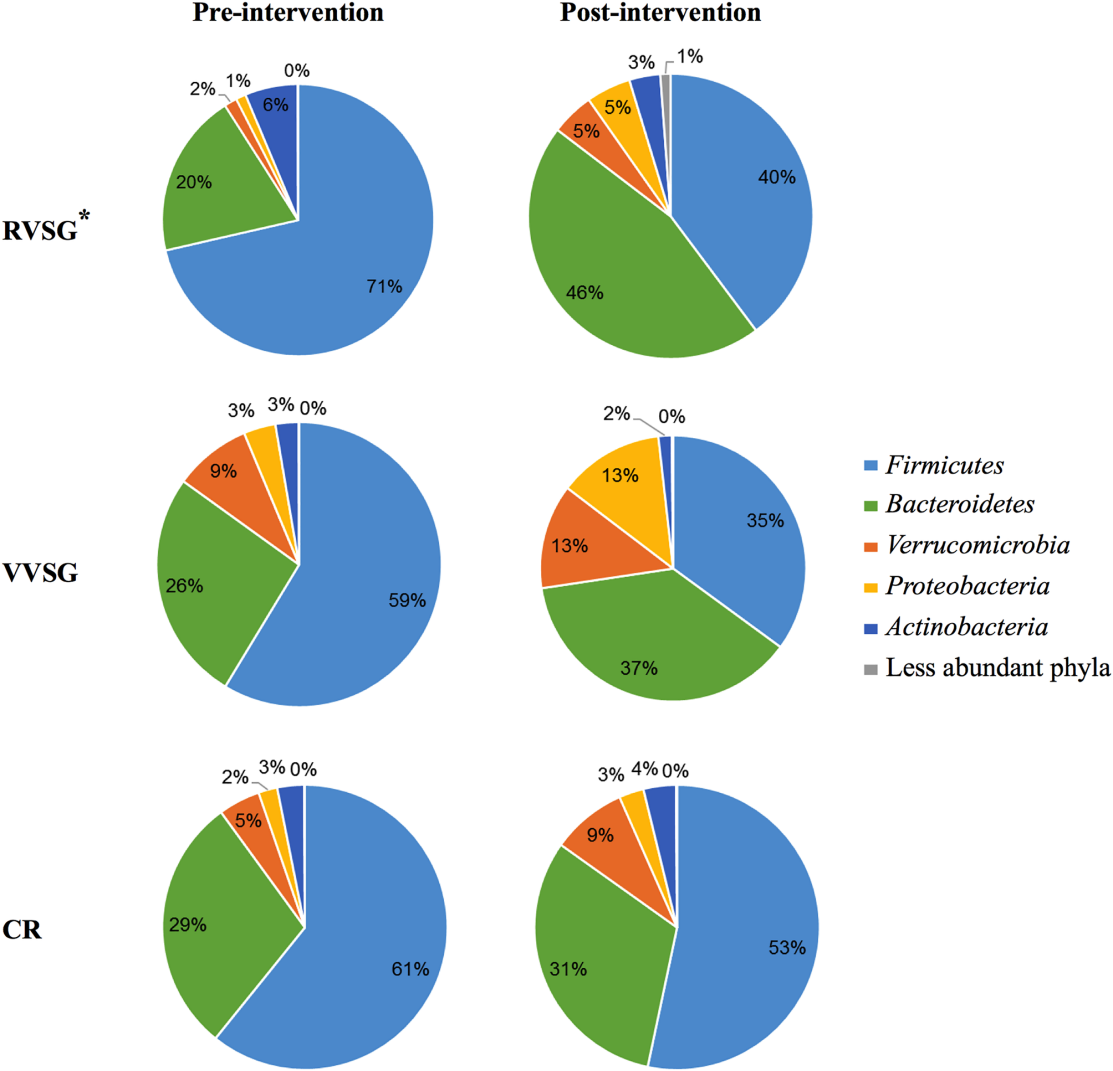
Pre- and post-intervention distribution of phyla averaged among all patients undergoing RVSG, VVSG, CR. *Indicates significant changes (*p* ≤ 0.01) in both *Firmicutes* and *Bacteroidetes*. Only RVSG post-intervention samples had significantly different community composition (beta diversity) from those of pre-intervention samples (*P* < 0.001).

**Figure 2:**
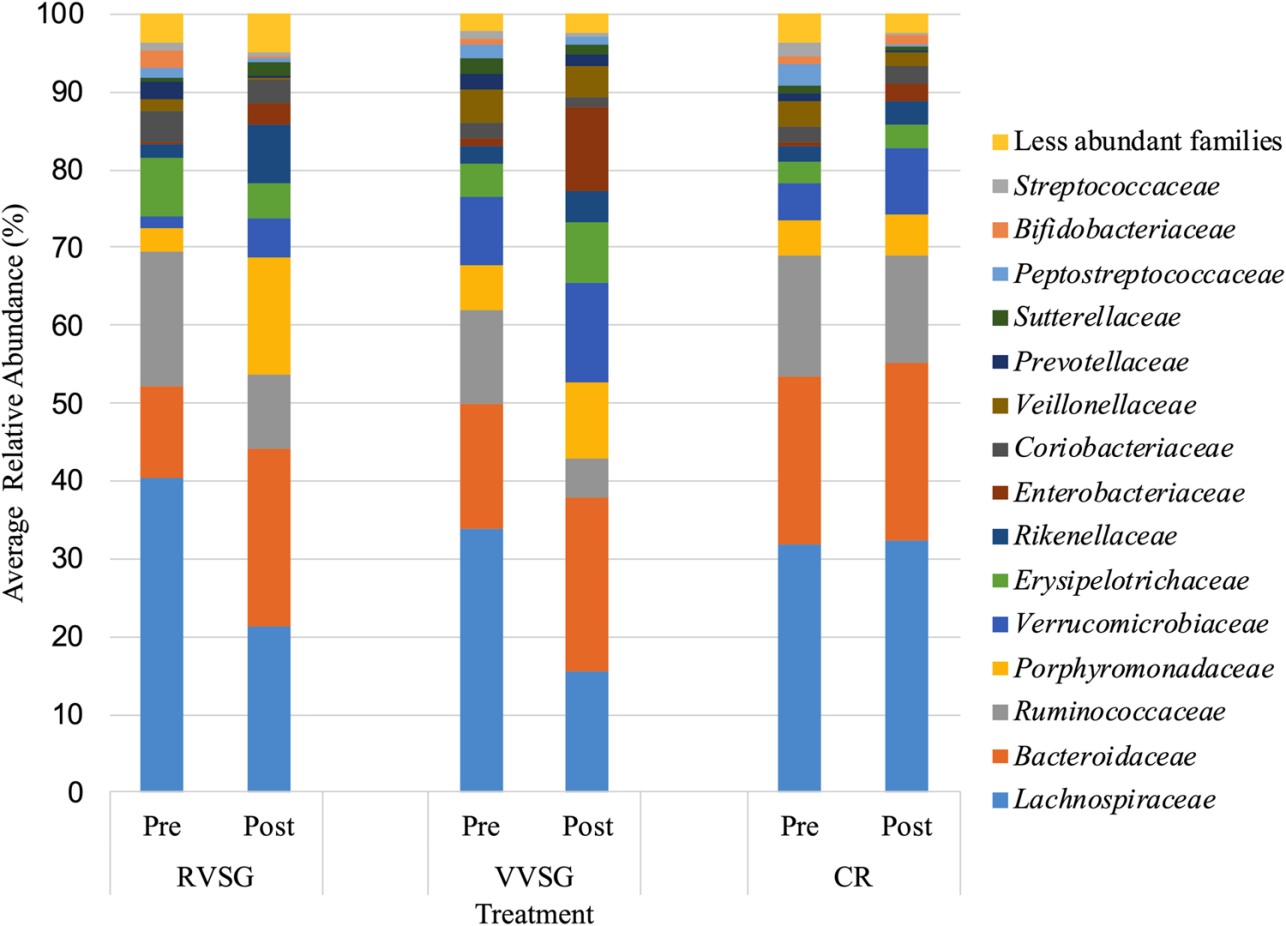
Distribution of family averaged among all patients within treatment groups (RVSG, VVSG, and CR) prior to and post-intervention. There was a significant reduction in members of the *Lachnospiraceae* family (*P* = 0.034) and an expansion in members of the *Porphyromonadaceae* family (*P* < 0.0001) among patients in the RVSG cohort. In VVSG, a reduction of *Streptococcaceae* (post-hoc* P* = 0.013) was observed, while no significant changes were seen in CR.

Some, but not all, studies have found an increased abundance of *Firmicutes* relative to *Bacteroidetes* in obese individuals^6,21^. Pre-intervention bacterial communities in our study were comparable between all groups, and were similarly dominated by members of the families *Lachnospiraceae* (29.2 ± 2.1% of sequence reads) and *Ruminococcaceae* (12.2 ± 1.1%), within the phylum *Firmicutes*, and members of the and *Bacteroidaceae* (19.3 ± 1.5%) and *Porphyromonadaceae* (7.4 ± 0.9%) within the *Bacteroidetes* phylum (Fig. 1). The relative abundances of *Bacteroidetes* to *Firmicutes* increased post-operatively following RVSG. However, the changes in relative abundances of these phyla were only statistically significant in the RVSG treatment arm (Fig. 1; *P* = 0.005 and 0.01, with respect to phyla). These changes among RVSG bacterial communities corresponded to a significant reduction in members of the *Lachnospiraceae* family (*P* = 0.034) and expansion in members of the *Porphyromonadaceae* family (*P* < 0.0001; Fig. 2). The reduction of *Streptococcaceae* in post-intervention VVSG samples was significant (Fig. 2; post-hoc* P* = 0.013). However, no significant shifts between pre- and post-intervention bacterial communities in the CR arm were observed.

### Comparison of pre- and post-intervention samples

**Figure 3:**
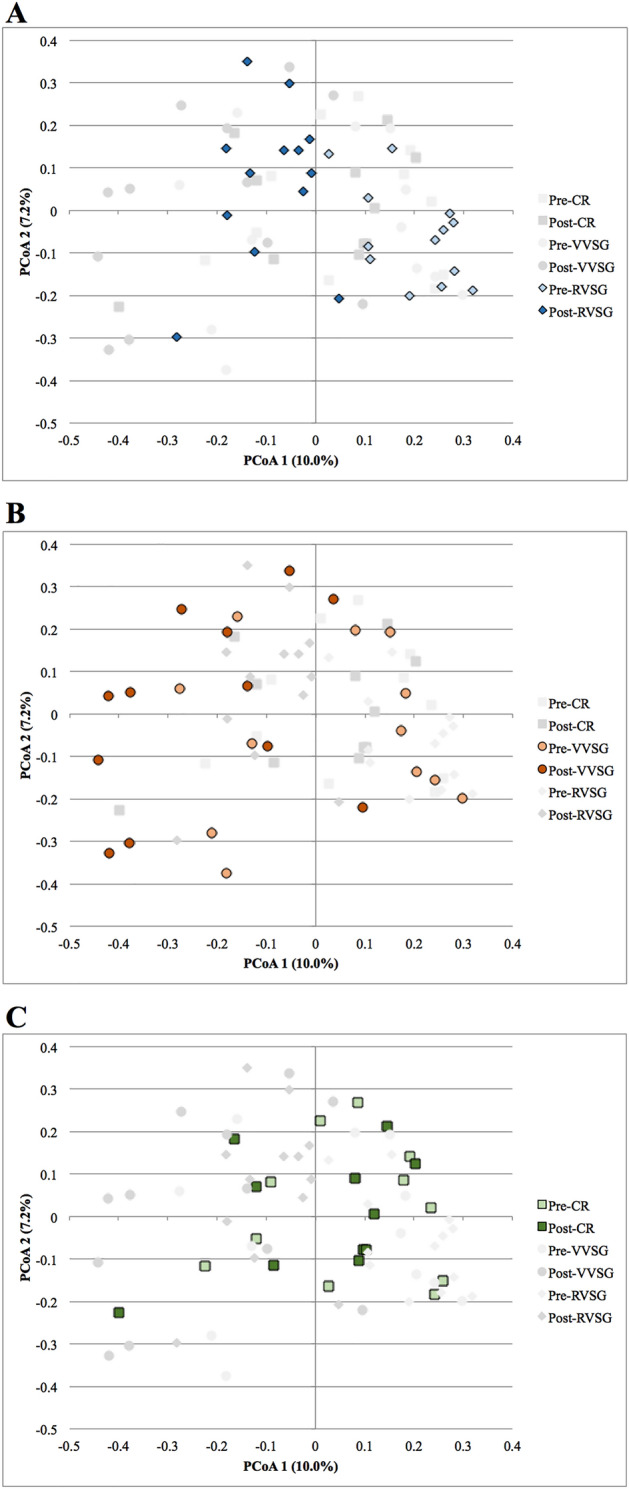
Principal coordinate analysis of (**A**) RVSG, (**B**) VVSG, and (**C**) CR samples pre- and post-intervention. Only post-operative communities in the RVSG cohort were significantly separated from all pre-operative communities (*p* < 0.001).

Only RVSG post-intervention samples had significantly different community composition (beta diversity) from those of pre-intervention samples (*P* < 0.001). Ordination of samples by PCoA and subsequent AMOVA analysis also indicated that only post-operative microbial communities for the RVSG arm were significantly separated from all pre-intervention microbial communities (Fig. 3A and Supplemental Figs. 1–3; *P* < 0.001). Excluding the two RVSG patients who received clindamycin did not significantly alter the results. Neither VVSG (Fig. 3B) nor CR (Fig. 3C) resulted in significant separation of pre- and post-intervention microbial communities.

The relationship between the structure and function of gut microbial communities has been shown in some cases. Specifically, changes in gut microbiota have been shown to be closely linked to alterations in bile acid composition^22,23^. Consequently, we measured the concentrations of fecal bile acids in all samples prior to and post-intervention (Supplemental Fig. 4). CR resulted in a significant reduction in deoxycholic acid (*P* = 0.01) and a trend towards reduced chenodeoxycholic acid (*P* = 0.15). There were no significant differences in measured bile acids following RVSG or VVSG, however there was a trend towards reduced levels of chenodeoxycholic acid (p = 0.16) in RVSG and a trend towards increased glycine conjugated bile acids following VVSG.

In an attempt to identify early microbiome markers predictive of weight loss, we examined Spearman correlations between changes in the relative abundances of families at 1-week post RVSG or VVSG and weight loss at 3 months post-surgery. An increase in relative abundance of *Bacteroidaceae* was strongly correlated with greater percent total weight loss (r = -0.82; *P* = 0.003). This finding was restricted only to the VVSG arm with no significant correlations identified in RVSG.

## Discussion

Changes in the composition of the gut microbiota following bariatric surgery have been causally linked to weight loss and metabolic improvement^14,24,25^. The aim of this study was to evaluate the relative contributions of the peri-operative antibiotics and caloric restriction to the postsurgical changes in the intestinal microbiota acutely following VSG prior to substantial weight loss. Routine antibiotics had a significant impact on microbial composition. When routine antibiotics were replaced with intravenous vancomycin, which has poor intestinal penetration, we observed no statistically shifts in the post-operative gut microbiome. Furthermore, post-operative caloric restriction alone comprised of a liquid diet with a stringent 800 kcal daily limit for 7 days did not have an appreciable effect on the gut microbiome but did alter fecal concentrations of deoxycholic acid. Taken together, our findings indicate that peri-operative antibiotics, and not the surgical procedure or caloric restriction per se, drive much of the early changes in the intestinal microbiota observed following VSG.

Our observations do not exclude the likely possibility that the VSG anatomy will impact the composition of the intestinal microbiota over time. We have previously identified an acute and sustained reduction in the relative abundance of *Firmicutes* and expansion of *Bacteroidetes* in a murine model of VSG^9^. Furthermore, VSG also results in altered gastrointestinal transit, reduced gastric acid secretion, faster gastric emptying, and shorter intestinal transit time, which are all factors expected to impact the intestinal microbiota. Thus, we expected to see shifts in the intestinal microbiota composition as well as in the fecal bile acid profile at this early time point. It is likely then that changes in the microbiome without the acute impact of antibiotics occur later than the 1-week time point at which we evaluated^26^. Importantly, identifying the significant impact that a single dose of an intestinal-penetrating antibiotic has on the post-surgical composition of the microbiota has potentially important implications longitudinally^19^. Our observations in the RVSG cohort appear similar to those seen by others at 3, 6 and 12 months post-VSG, specifically with a decrease in the relative abundance of *Firmicutes* and expansion of *Bacteroidetes*^27–29^. Antibiotic administration, which can have long-lasting effects on the composition of the indigenous microbiota for months and even years following short-term use, has gone undocumented^12,24,30–32^. We recently showed in a mouse model of VSG that disrupting the postsurgical shift in microbial composition with antibiotics acutely following surgery not only results in less weight loss, but importantly the loss of any metabolic benefit. Interestingly, in this study we identified persistent changes in the composition of the microbiome 1 month after the last dose of antibiotic was administered highlighting a longitudinal impact. Whether these observations are translated in humans following surgery is unknown at this time but is an important next step that we are investigating. It is noteworthy that studies are supporting a role for post-surgical microbial changes in predicting the resolution of T2DM^12,33^. Observations from our study suggest that antibiotics have the potential to diminish or even eliminate such potentially metabolically important changes. For example, we observed a significant reduction in the relative abundance of *Lachnospiraceae* family only following RVSG and was not seen in the VVSG or CR, supposing that this is an antibiotic-specific change. Davies et al. have shown that the expansion of *Lachnospiraceae* was more abundant in those who achieved diabetes remission 1 year following bariatric surgery, highlighting a potentially negative impact of routine antibiotic administration^33^. Furthermore, our observation on the correlation of *Bacteroidaceae* and weight loss in the VVSG arm is noteworthy as a greater abundance of *Bacteroidaceae* has been correlated to resistance to diet-induced obesity and a healthier metabolic profile^34,35^.

Changes in the composition of the gut microbiome have been shown to be closely linked to alterations in bile acids^22,23^. We previously observed in humans that serum glycine-conjugated, unconjugated, and secondary bile acids increased 1 week following VSG^36^. However, no significant differences were observed in fecal bile acids in either VSG group. It is plausible that more rapid delivery of bile acids to the distal gut may facilitate an increase in serum bile acid concentrations, although more studies are required to confirm this possibility. We did observe a reduction in fecal deoxycholic acid in the CR cohort which matches our previously published serum studies in a different CR group^36^. Thus, these results suggest that CR may act in a manner that is distinct from VSG and CR together.

There are some important limitations to our study. We assessed early changes in fecal microbiota following surgery. Future studies with a larger sample size are needed to correlate these results with long-term microbiome metrics, and any potential relationship with long-term metabolic outcomes. While weight loss did not significantly differ among the three cohorts we examined, our study is limited by a lack of detailed metabolic testing. This is an important next step in characterizing metabolic changes, such as insulin resistance, in concert with changes in the intestinal microbiota. Although previous investigations showed minimal intestinal penetration of IV vancomycin, it is possible that there may be some impact given our finding of reduced alpha diversity following VVSG^37^. We profiled fecal microbiome, but realize that it may not be a sufficient surrogate for small bowel and/or mucosa-adherent intestinal microbiome that may have greater relevance to energy metabolism^38,39^. Finally, we did not control for the potential impact(s) of bowel prep, anesthesia and short-term narcotic use on the intestinal microbiota in the CR cohort. These other potential confounders may be included in future studies to further isolate VSG driven effects.

In conclusion, the results of this study indicate that the immediate post-operative shift in the intestinal microbial community structure following VSG is significantly impacted by the single dose of antibiotic administration and not by caloric restriction or the resultant anatomic changes. This study adds to the accumulating data highlighting the potentially adverse effects of antibiotic induced dysbiosis on metabolic health. Moreover, it raises important questions with regards to the impact of VSG on the gut microbiota, the necessity of peri-operative antibiotics, and the type of antibiotics that should be administered to patients at the time of surgery.

## Patients and methods

### Study subjects

Institutional Review Board (IRB) approval was obtained from St. Cloud Hospital, Minnesota, for study protocols. All experiments were performed in accordance with relevant named guidelines and regulations. All patients under consideration for this study were > 18 years of age with a body mass index (BMI) ≥ 35.0 kg/m^2^ (Table 1). Patients with a previous history of any gastrointestinal surgery, including cholecystectomy, or recent antibiotic use (within 3 months of initiation of intervention), were excluded from the study.

### Experimental design

**Figure 4:**
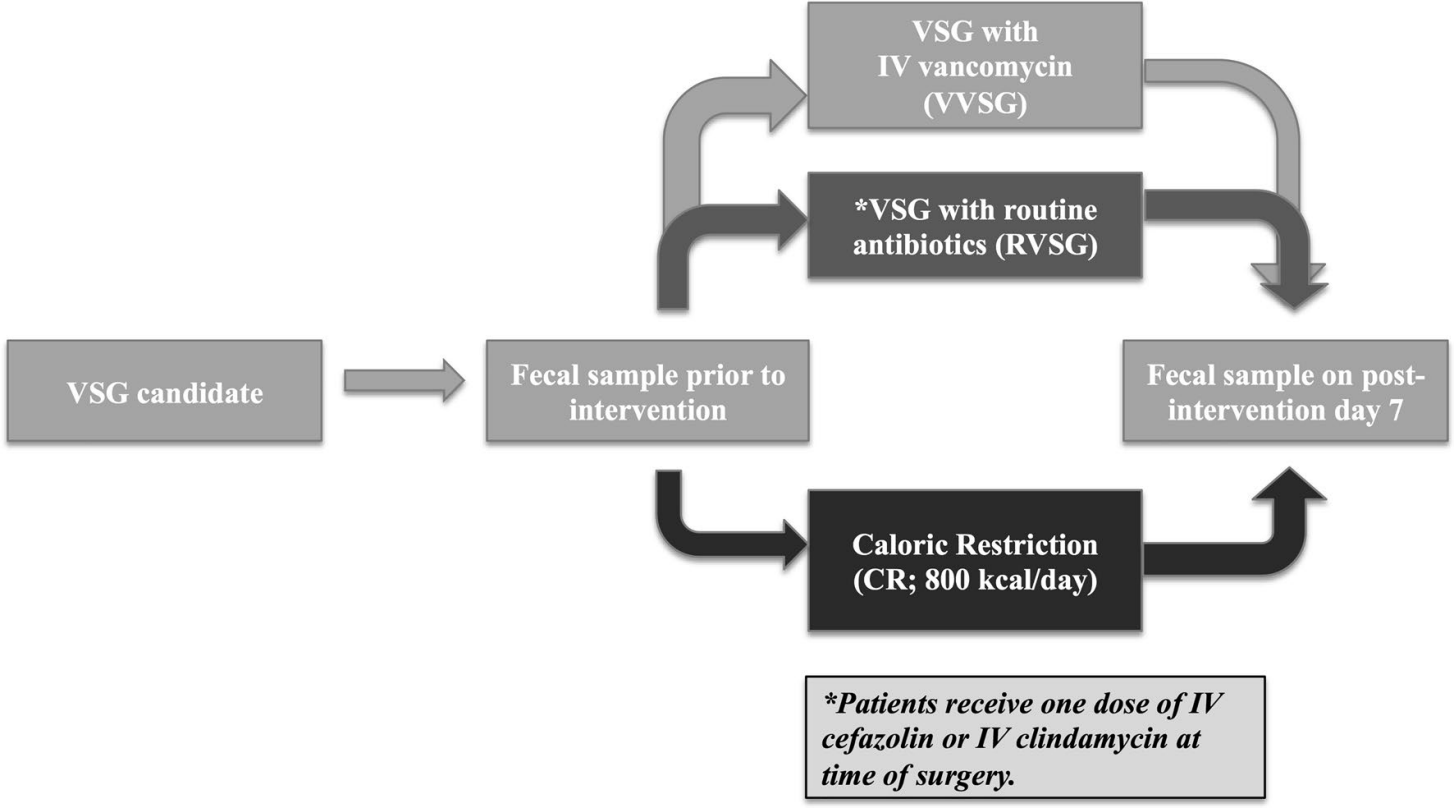
Study design. The objective of the study was to evaluate the relative contributions of important peri-operative factors in shaping the gut microbiome acutely following VSG in order to identify changes that may contribute to the metabolic efficacy of the procedure. 32 patients were recruited to undergo VSG with routine intravenous antibiotics (RVSG; n = 12), VSG with intravenous vancomycin as peri-operative antibiotic (VVSG; n = 12), or caloric restriction (CR; n = 8) which is routinely implemented in post-surgical cares. Intravenous vancomycin was specifically chosen due to its poor intestinal penetrance thus allowing for the evaluation of the impact of the single dose of peri-operative antibiotic on the composition of the microbiome.

The three arms of the study were sequentially blocked concurrent with recruitment (Fig. 4). Twelve consecutive patients received routine antibiotics (RVSG) immediately prior to surgery that consisted of 2 g of IV cefazolin (n = 10) or 600 mg of IV clindamycin (n = 2) 600 mg in the case of a penicillin allergy. Twelve consecutive patients underwent VSG and received a 1500 mg dose of IV vancomycin administered immediately prior to surgery (VVSG). Eight consecutive patients not undergoing surgery were placed on a calorically restricted (CR) diet matching that which post-bariatric surgery patients adhere.

All patients were placed on identical diets consisting of four clear liquid Robard (Mt. Laurel, NJ) protein shakes totaling 760–800 kcal a day for 6 days as per our standard dietary protocol at the time this study was being conducted. Nutritional content consisted of 24 g of fat, 102–108 g of protein, and 40–50 g of carbohydrates per day. Dietary plan and execution were performed under the guidance of a bariatric registered dietitian (KE).

### Surgery

Briefly, laparoscopic VSG was performed as follows: a 4.8 mm stapler load was used to divide the greater curvature of the stomach 6 cm from the pylorus and remaining 3 cm from the *angularis incisura*. A 34 French Bougie was inserted and guided the first staple firing. Multiple 3.5 mm stapler loads were fired thereafter progressing up to the angle of His to complete VSG. A Foley catheter was not inserted and preoperative skin preparation was performed with chlorhexidine.

### Sample collection

All patients were given instructions and stool collection materials at their final pre-intervention clinic visit. Participants were asked to collect a fecal sample prior to initiation of intervention and the day prior to their first post-operative visit (POD 6 or post-diet day 6). Patients stored samples in their home freezers for up to 24 h. Upon delivery to the laboratory, samples were kept frozen at − 80 °C until processed.

### Sample processing and sequencing

DNA was isolated from human stool samples using Powersoil DNA Isolation Kits (MO BIO Laboratories, #12888-100) per manufacturer’s instructions. The V5 + V6 hypervariable regions of the 16S rRNA gene were amplified using the BSF 784/1046R primer set (CS1). Amplicons were gel purified and pooled in equal concentrations. Sample libraries were paired-end sequenced at a read length of 300 nt on the Illumina MiSeq platform by the University of Minnesota Genomics Center (Minneapolis, MN). Raw data was received as fastq files and are deposited in the National Center for BioTechnology Information Sequence Read Archive under BioProject accession number SRP072183.

All sequence processing and analysis was performed using mothur software ver. 1.34.0^40^. Raw sequence data was trimmed to 150 nt for both forward and reverse reads to remove low-quality regions at the end of sequence reads. Trimmed reads were paired-end joined using fastq-join software^41^. Joined reads where then trimmed for quality using the following criteria: average quality scores ≥ 35 over a window of 50 nt, homopolymers ≤ 8 nt, no ambiguous bases, and ≤ 2 mismatches from primer sequences. Sequence reads were aligned against the SILVA database ver. 119^42^ and subjected to a 2% pre-clustering step to remove sequence errors^43^. Chimeras were rarefied and removed using UCHIME software^44^. For comparisons, each sample was normalized, by random subsampling to 25,000 sequences. Operational taxonomic units (OTUs) were assigned at 97% similarity using the furthest-neighbor algorithm, and taxonomic classifications were performed against the Ribosomal Database Project database ver. 14^45^.

### Statistical analysis

Alpha and beta diversity indices and ordination analysis were performed using mothur. The Shannon index was calculated to provide a parametric measure of alpha, or species, diversity. The Kruskal–Wallis test was used to determine significant variations in OTUs among treatment groups^46^. Beta diversity comparisons (community composition) were performed using Bray–Curtis dissimilarity matrices analysis of similarity (ANOSIM)^47,48^. Analysis of molecular variance (AMOVA)^49^ was also performed to statistically determine sample clustering, and ordination was performed via principal coordinate analysis (PCoA).

Bray–Curtis matrices are plotted using PCoA, which allows simple visualization of similarities among compositions of different samples given the large complex data obtained from sequencing. Spearman correlations were performed using XLSTAT ver. 2015.01.0 (Addinsoft, Belmont, MA). Data with p-values less than 0.05 were considered statistically significant, unless otherwise stated, and the Bonferroni correction was applied to Spearman rank correlation values to correct for multiple comparisons. A power analysis was performed using the HMP package in R ver. 3.2.2. A Monte-Carlo simulation procedure was used to estimate the power of finding differences in abundances of phyla using a rank abundance distribution probability test^50^.

The power (beta) of each arm to detect differences in post-intervention communities at the sample sizes used in this study was 1.00 for all arms.

### Bile acid measurements

Fecal composition was determined on all samples as previously described as follows^51^. Fecal samples were added to 10 vol (w/v) of 50% aqueous acetonitrile with 5 μM oleanolic acid (internal standard) and extracted by vortex and sonication for 10 min. The mixture was centrifuged twice at 18,000×*g* for 10 min, the supernatant was transferred into a sample vial and subjected to LC–MS analysis. A 5 µl aliquot of fecal extract was injected into an Acquity UPLC system (Waters, Milford, MA) and separated using Water Acquity C18 column (1.7 μm, 2.1 × 50 mm). Mobile phases A and B were water with 10uM ammonium acetate at pH9 and 95% acetonitrile, 5% water, with 10 mM ammonium acetate at pH 9, respectively. The mobile phase gradient ranged from 0.5 to 100% B over a 10-min run. The LC eluent was introduced into a Xevo-G2-S QTOF mass spectrometer (Waters) for metabolite identification and quantification. Capillary and cone voltage for electrospray ionization (ESI) were maintained at − 10 V and − 5 V for negative-mode detection, respectively. Source temperature and desolvation temperature were 120 °C and 350 °C, respectively. Nitrogen was used as a cone (50 l/h) and desolvation gas (800 l/h). For accurate mass measurement, the mass spectrometer was calibrated with a sodium formate solution (range *m/z* 50–1000) and monitored by the intermittent injection of the lock mass leucine enkephalin ([M−H]^−^ = 554.2615 m*/z*) in real time. Mass chromatograms and mass spectral data were acquired and processed by MassLynx software (Waters) in centroided format. The concentrations of various primary (cholic acid and chenodeoxycholic acid), secondary bile acids (deoxycholic acid and lithocholic acid), taurine-conjugated bile acids (taurocholic acid, taurochenodeoxycholic acid, and taurodeoxycholic acid), and glycine-conjugated bile acids (glycodeoxycholic acid, glycocholic acid, and glycochenodeoxcholic acid) in serum samples were determined using corresponding standard curves and QuanLynx software (Waters)^51^.

### Ethics approval

All study procedures were in accordance with the ethical standards of the national research ethics committee and with the 1964 Helsinki declaration and its later amendments or comparable ethical standards.

### Informed consent

Informed consent was obtained from all individual participants for whom identifying information is included in this article.

## Supplementary information

Supplementary Figures.

Supplementary Captions.

## References

[1] Ikramuddin S (2012). Roux-en-Y gastric bypass versus intensive medical management for the control of type 2 diabetes, hypertension and hyperlipidemia: an international, multicenter randomized trial. JAMA.

[2] Schauer PR (2014). Bariatric surgery versus intensive medical therapy for diabetes—3-year outcomes. N. Engl. J. Med..

[3] Sjöström L (2009). Effects of bariatric surgery on cancer incidence in obese patients in Sweden (Swedish Obese Subjects Study): a prospective, controlled intervention trial. Lancet Oncol..

[4] Abraham A (2016). Trends in bariatric surgery: procedure selection, revisional surgeries, and readmissions. Obes. Surg..

[5] Ikramuddin S (2013). Roux-en-Y gastric bypass vs intensive medical management for the control of type 2 diabetes, hypertension, and hyperlipidemia the diabetes surgery study randomized clinical trial. JAMA.

[6] Turnbaugh PJ (2006). An obesity-associated gut microbiome with increased capacity for energy harvest. Nature.

[7] Ridaura VK (2013). Cultured gut microbiota from twins discordant for obesity modulate adiposity and metabolic phenotypes in mice. Science.

[8] Ryan KK (2014). FXR is a molecular target for the effects of vertical sleeve gastrectomy. Nature.

[9] Jahansouz C (2017). Sleeve gastrectomy drives persistent shifts in the gut microbiome. Surg. Obes. Relat. Dis..

[10] Mcgavigan AK (2015). TGR5 contributes to glucoregulatory improvements after vertical sleeve gastrectomy in mice. Gut.

[11] Liou AP (2013). Conserved shifts in the gut microbiota due to gastric bypass reduce host weight and adiposity. Sci. Transl. Med..

[12] Murphy R (2017). Differential changes in gut microbiota after gastric bypass and sleeve gastrectomy bariatric surgery vary according to diabetes remission. Obes. Surg..

[13] Sánchez-Alcoholado L (2019). Gut microbiota adaptation after weight loss by Roux-en-Y gastric bypass or sleeve gastrectomy bariatric surgeries. Surg. Obes. Relat. Dis..

[14] Tremaroli V, Karlsson F, Werling M, Roux CW, Kovatcheva-datchary P (2015). Roux-en-Y gastric bypass and vertical banded gastroplasty induce long-term changes on the human gut microbiome contributing to fat mass regulation. Cell Metab..

[15] Coluzzi I (2016). Food intake and changes in eating behavior after laparoscopic sleeve gastrectomy. Obes. Surg..

[16] David LA (2014). Diet rapidly and reproducibly alters the human gut microbiome. Nature.

[17] Halsall AK, Welsh CL, Craven JL, Hopton DS, Peel RN (1980). Prophylactic use of metronidazole in preventing wound sepsis after elective cholecystectomy. Br. J. Surg..

[18] Cho I (2013). Antibiotics in early life alter the murine colonic microbiota and adiposity. Nature.

[19] Jahansouz C (2019). Antibiotic-induced disruption of intestinal microbiota contributes to failure of vertical sleeve gastrectomy. Ann. Surg..

[20] Moellering RC (1984). Pharmacokinetics of vancomycin. J. Antimicrob. Chemother..

[21] Ley RE (2010). Obesity and the human microbiome. Curr. Opin. Gastroenterol..

[22] Sayin SI (2013). Gut microbiota regulates bile acid metabolism by reducing the levels of tauro-beta-muricholic acid, a naturally occurring FXR antagonist. Cell Metab..

[23] Wahlström A, Sayin SI, Marschall H-U, Bäckhed F (2016). Intestinal crosstalk between bile acids and microbiota and its impact on host metabolism. Cell Metab..

[24] Damms-Machado A (2015). Effects of surgical and dietary weight loss therapy for obesity on gut microbiota composition and nutrient absorption. Biomed Res. Int..

[25] Palleja A (2016). Roux-en-Y gastric bypass surgery of morbidly obese patients induces swift and persistent changes of the individual gut microbiota. Genome Med..

[26] Melissas J (2008). Sleeve gastrectomy—a ‘food limiting’ operation. Obes. Surg..

[27] Graessler J (2012). Metagenomic sequencing of the human gut microbiome before and after bariatric surgery in obese patients with type 2 diabetes: correlation with inflammatory and metabolic parameters. Pharmacogenomics J..

[28] Zhang H (2009). Human gut microbiota in obesity and after gastric bypass. Proc. Natl. Acad. Sci. U.S.A..

[29] Kong L, Aron-Wisnewsky J, Pelloux V, Basdevant A, Bouillot J, Tap J, Zucker J, Dore J, Clement K (2013). Gut microbiota after gastric bypass in human obesity: increased richness and associations of bacterial genera with adipose tissue genes. Am. Soc. Nutr..

[30] Dethlefsen L, Relman DA (2011). Incomplete recovery and individualized responses of the human distal gut microbiota to repeated antibiotic perturbation. Proc. Natl. Acad. Sci. U.S.A..

[31] Jernberg C, Lö Fmark S, Edlund C, Jansson JK (2007). Long-term ecological impacts of antibiotic administration on the human intestinal microbiota. ISME J..

[32] Zaura E (2015). Same exposure but two radically different responses to antibiotics: resilience of the salivary microbiome versus long-term microbial shifts in feces. Am. Soc. Microbiol..

[33] Davies N, O’Sullivan JM, Plank LD, Murphy R (2020). Gut microbial predictors of type 2 diabetes remission following bariatric surgery. Obes. Surg..

[34] Hughes RL, Kable ME, Marco M, Keim NL (2019). The role of the gut microbiome in predicting response to diet and the development of precision nutrition models. Part II: results. Adv. Nutr..

[35] Kreznar JH (2017). Host genotype and gut microbiome modulate insulin secretion and diet-induced metabolic phenotypes. Cell Rep..

[36] Jahansouz C (2016). Bile acids increase independently from hypocaloric restriction after bariatric surgery. Ann. Surg..

[37] Currie BP, Lemos-Filho L (2004). Evidence for biliary excretion of vancomycin into stool during intravenous therapy: potential implications for rectal colonization with vancomycin-resistant enterococci. Antimicrob. Agents Chemother..

[38] Chen RY (2020). Duodenal microbiota in stunted undernourished children with enteropathy. N. Engl. J. Med..

[39] Martinez-Guryn K, Leone V, Chang EB (2019). Regional diversity of the gastrointestinal microbiome. Cell Host Microbe.

[40] Schloss PD (2009). Introducing mothur: open-source, platform-independent, community-supported software for describing and comparing microbial communities. Appl. Environ. Microbiol..

[41] Aronesty E (2013). Comparison of sequencing utility programs. Open Bioinform. J..

[42] Pruesse E (2007). SILVA: a comprehensive online resource for quality checked and aligned ribosomal RNA sequence data compatible with ARB. Nucleic Acids Res..

[43] Huse SM, Welch DM, Morrison HG, Sogin ML (2010). Ironing out the wrinkles in the rare biosphere through improved OTU clustering. Environ. Microbiol..

[44] Edgar RC, Haas BJ, Clemente JC, Quince C, Knight R (2011). UCHIME improves sensitivity and speed of chimera detection. Bioinformatics.

[45] Cole JR (2009). The Ribosomal Database Project: improved alignments and new tools for rRNA analysis. Nucleic Acids Res..

[46] Acar EF, Sun L (2013). A generalized Kruskal–Wallis test incorporating group uncertainty with application to genetic association studies. Biometrics.

[47] Clarke KR (1993). Non-parametric multivariate analyses of changes in community structure. Aust. J. Ecol..

[48] Bray JR, Curtis JT (1957). An ordination of the upland forest communities of Southern Wisconsin. Ecol. Monogr..

[49] Excoffier L, Smouse PE, Quattro JM (1992). Analysis of molecular variance inferred from metric distances among DNA haplotypes—application to human mitochondrial DNA restriction data. Genetics.

[50] la Rosa PS (2012). Hypothesis testing and power calculations for taxonomic-based human microbiome data. PLoS ONE.

[51] Weingarden AR (2014). Microbiota transplantation restores normal fecal bile acid composition in recurrent *Clostridium**difficile* infection. Am. J. Physiol. Gastrointest. Liver Physiol..

