# Supplementary figures and images for: Peri-operative antibiotics acutely and significantly impact intestinal microbiota following bariatric surgery
Source: Sci Rep. 2020 Nov 23;10:20340. doi: 10.1038/s41598-020-77285-7 (PMC7684314; doi:10.1038/s41598-020-77285-7)

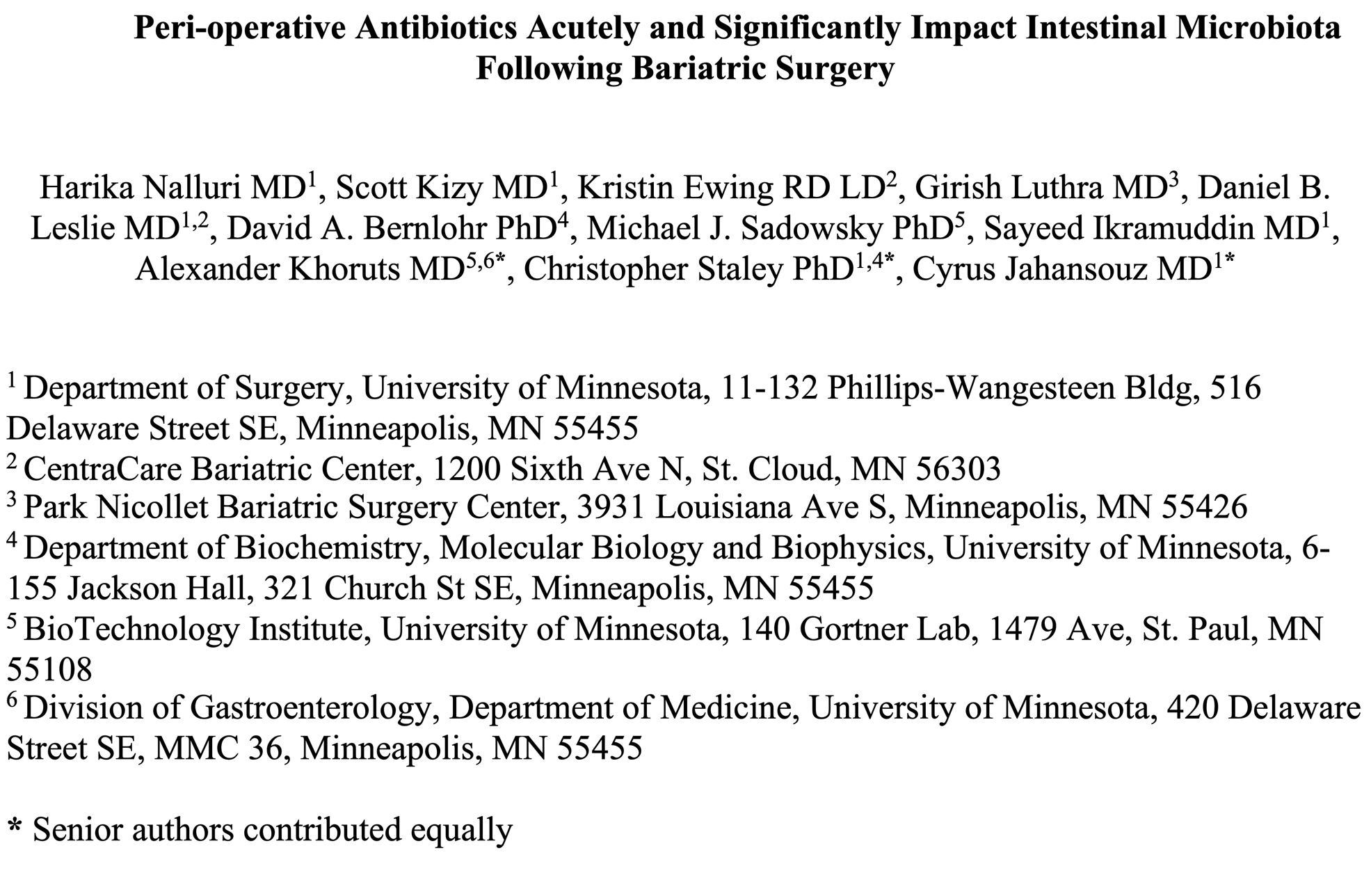

Supplement: Supplementary file 1 — Supplementary Figures. [file 41598_2020_77285_MOESM1_ESM.tiff]
