# Supplementary material for: Peri-operative antibiotics acutely and significantly impact intestinal microbiota following bariatric surgery
Source: Sci Rep. 2020 Nov 23;10:20340. doi: 10.1038/s41598-020-77285-7 (PMC7684314; doi:10.1038/s41598-020-77285-7)
Supplement: Supplementary file 2 — Supplementary Captions. [file 41598_2020_77285_MOESM2_ESM.docx]

**Supplemental Figure 1.** Principal coordinate analysis of all pre-intervention samples and VSG with routine peri-operative intravenous antibiotics (RVSG) post-intervention samples.

**Supplemental Figure 2.** Principal coordinate analysis of all pre-intervention samples and VSG with intravenous vancomycin (VVSG) post-intervention samples.

**Supplemental Figure 3.** Principal coordinate analysis of all pre-intervention samples and caloric restriction (CR) post-intervention samples.

**Supplemental Figure 4.** Pre- and post- intervention concentrations of bile acids averaged among all patients: (A) Cholic Acid, (B) Chenodeoxycholic Acid, (C) Deoxycholic Acid, (D) Lithocholic Acid, (E) Glycine Conjugated bile acids, (F) Taurine Conjugated bile acids. Fecal levels of deoxycholic acid significantly decreased following caloric restriction. Values are mean ± SEM; * p < 0.05.
